# P-1590. Public Health and Economic Impact of Increased Uptake of an Additional Dose of Pfizer-BioNTech KP.2-Adapted COVID-19 Vaccine, 2024-2025 Formula, Among US Adults ≥ 65 Years of Age

**DOI:** 10.1093/ofid/ofaf695.1769

**Published:** 2026-01-11

**Authors:** Alon Yehoshua, Manuela Di Fusco, Abby Rudolph, Elizabeth A Thoburn, Santiago M C Lopez, Ben Yarnoff

**Affiliations:** Pfizer Inc., New York, New York; Pfizer Inc, New York, New York; Pfizer Inc, New York, New York; Pfizer, Inc, Collegeville, PA; Pfizer Inc, New York, New York; PPD Clinical Research Services, Thermo Fisher Scientific, Siler CIty, North Carolina

## Abstract

**Background:**

Despite ACIP recommendation that United States (US) adults ≥ 65 years of age receive an additional dose of the COVID-19 vaccine, uptake remains low. This study aimed to estimate the public health and economic impact of increased uptake of an additional dose of Pfizer-BioNTech KP.2-adapted COVID-19 vaccine, 2024-2025 formula, in US adults aged ≥ 65 years.

**Table 1.**
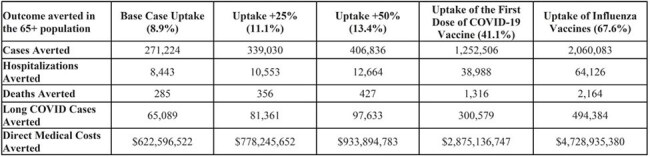
COVID-19 Cases, Hospitalizations, Deaths, Long COVID Cases, and Direct Medical Costs Averted by an Additional Dose of Pfizer-BioNTech KP.2-adapted COVID-19 vaccine, 2024-2025 Formula, by Uptake

**Methods:**

A previously described decision tree model was adapted to estimate the public health and economic impact of increasing the uptake of an additional dose by US adults aged ≥ 65 years over a one-year time horizon. The base case assumed the same vaccine uptake observed for an additional dose in the 2024-2025 season (8.9%) and 4 alternative hypothetical uptake scenarios were examined with wide ranges (+25%, +50%, same as initial dose [41.1%], same as influenza [67.6%]). Inputs specific to adults aged ≥ 65 years for the monthly probability of infection, annual probability of hospitalization, and annual vaccine uptake were informed by public health surveillance data. Other inputs for clinical probabilities, cost, and vaccine effectiveness were informed by literature. Conservatively, indirect effects of vaccination on reduced community transmission were not considered.

**Results:**

In the base case, an additional dose of Pfizer-BioNTech KP.2-adapted COVID-19 vaccine in adults aged ≥ 65 years was estimated to prevent 271,224 cases, 8,443 hospitalizations, 285 deaths, 65,089 long COVID cases, and $623 million in direct medical costs. Increasing uptake to 11.1% (+25%), 13.4% (+50%), and to 41.1% (the same as the initial dose), and 67.6% (the same as influenza vaccines) would increase the estimated public health and economic impact (Table 1). For example, increasing uptake from 8.9% to 41.1% was estimated to increase hospitalizations averted to 38,988, deaths averted to 1,316, and direct medical costs to $2.8 billion (Table 1).

**Conclusion:**

Increasing uptake of an additional dose of Pfizer-BioNTech KP.2-adapted COVID-19 vaccine was estimated to substantially reduce the burden of COVID-19 among US adults aged ≥ 65 years. Future vaccine policy decisions can focus on increasing uptake of the additional dose.

**Disclosures:**

Alon Yehoshua, PharmD, MS, Pfizer Inc: Employee of Pfizer and may hold stock or stock options of Pfizer Manuela Di Fusco, PhD, Pfizer Inc: Employee of Pfizer and may hold stock or stock options of Pfizer Abby Rudolph, PhD, Pfizer Inc: Employee of Pfizer and may hold stock or stock options of Pfizer Elizabeth A. Thoburn, MPH, Pfizer Inc: Employee of Pfizer and may hold stock or stock options of Pfizer Santiago M.C. Lopez, MD, Pfizer Inc.: Employee of Pfizer Inc. and may hold stock or stock options|Pfizer Inc.: Stocks/Bonds (Public Company) Ben Yarnoff, PhD, Pfizer Inc: An employee of Thermo Fisher Scientific, which received funding from Pfizer in connection with the study and the development of this abstract

